# Data for the effect of histone deacetylase inhibitors on voltage- and ligand-gated ion channel gene expression in neurogenic induced-human adipose tissue-derived mesenchymal stem cells

**DOI:** 10.1016/j.dib.2018.02.058

**Published:** 2018-02-27

**Authors:** Sujeong Jang, Han-Seong Jeong

**Affiliations:** Department of Physiology, Chonnam National University Medical School, Gwangju 61469, Republic of Korea

**Keywords:** Histone deacetylase inhibitors, Stem cells, Cell differentiation, Ion channels, Neurofilament-L

## Abstract

This data article contains descriptive and experimental data on ion channel gene expressions following the histone deacetylase (HDAC) inhibitor treatment of neural induced human adipose tissue-derived mesenchymal stem cells (NI-hADSCs). Following treatment of the HDAC inhibitors, such as MS-275, NaB, TSA, or VPA, the phenotypes of NI-hADSCs exhibit neuron-like features and the neurofilament-L (NFL)-positive cells were increased. The expression of the ion channel marker genes, such as *SCN5A*, *KCNA4*, and *CACNA1G*, was highly increased following treatment with the HDAC inhibitors; however, the expression of others was either decreased or unchanged. For further details and experimental findings please refer to the research article by Jang and Jeong. Histone deacetylase inhibition-mediated neuronal differentiation via the Wnt signaling pathway in human adipose tissue-derived mesenchymal stem cells (Jang and Jeong, 2018) [1].

**Specifications Table**

**Table:** 

Subject area	*Biology*
More specific subject area	*Stem cell differentiation*
Type of data	*Table, Graphs, Figures*
How data was acquired	*Phase Contrast Microscopy (Axio Vert. A1, Carl Zeiss, Germany), Immunofluorescence staining*
	*RT-PCR analysis (Takara Bio Inc., Shiga, Japan)*
Data format	*Raw and analyzed data*
Experimental factors	*Neural induction: 100* *ng/mL of basic fibroblast growth factor (bFGF) and 10 μM of forskolin*
	*Histone deacetylase inhibitors: MS-275 (500 nM), Sodium butyrate (NaB, 10 μM), Trichostatin A (TSA, 40 μM), Valproic acid (VPA, 10 μM)*
Experimental features	*The effect of HDAC inhibitors in neurogenic differentiation, which is shown as a neurofilament-L positive, was analyzed following the expression of ion channel-related gene.*
Data source location	*Stem Cell and Regenerative Medicine Laboratory, Department of Physiology, Chonnam National University Medical School, Gwangju, Republic of Korea*
Data accessibility	*The data are available with this article.*

**Value of the data**

• This method can be used to control stem cell fate and develop the neurogenic differentiation of the cells.
• Phenotypes of NI-hADSCs exhibited distinct neuron-like morphologies with branched processes following HDAC inhibitor treatment.
• NFL expressed cells were increased with HDAC inhibitor treatment.
• The ion channel marker genes were upregulated following treatment with the HDAC inhibitors.

## 1 Data

**Fig. 1:**
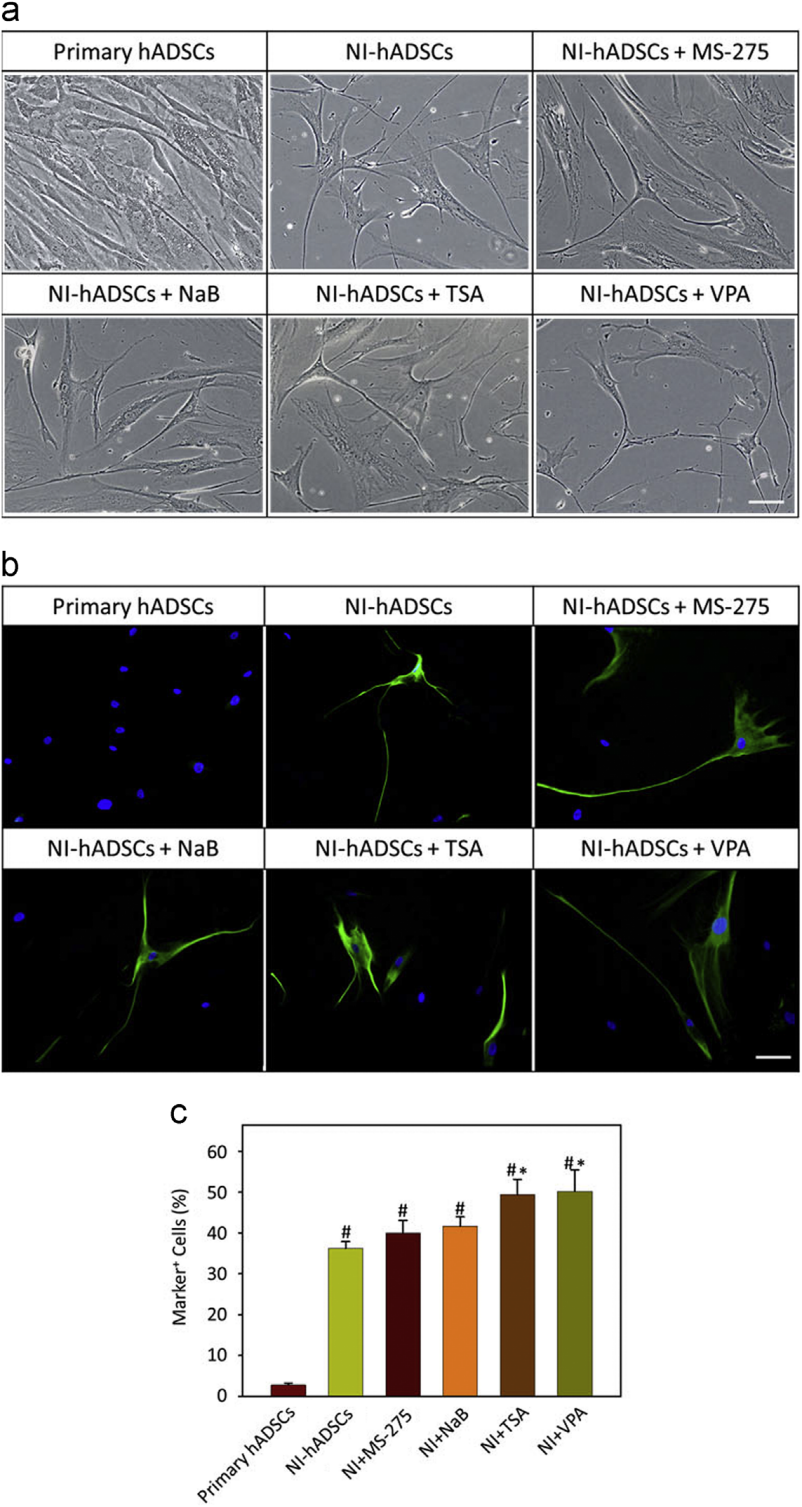
The morphology and fluorescent immunocytochemistry of hADSCs after treatment with HDAC inhibitors. (a) Phase contrast microscopy. (b) Immunofluorescence of NFL. Scale bars equate to 20 μm. (c) Histograms show quantification of fluorescence intensity of NFL. Data represent the mean±SEM; *n*=3 (#*p*<0.05 compared with the ratio from primary hADSCs, *p<0.05 compared with the ratio from NI-hADSCs without HDAC inhibitor treatment).

**Fig. 2:**
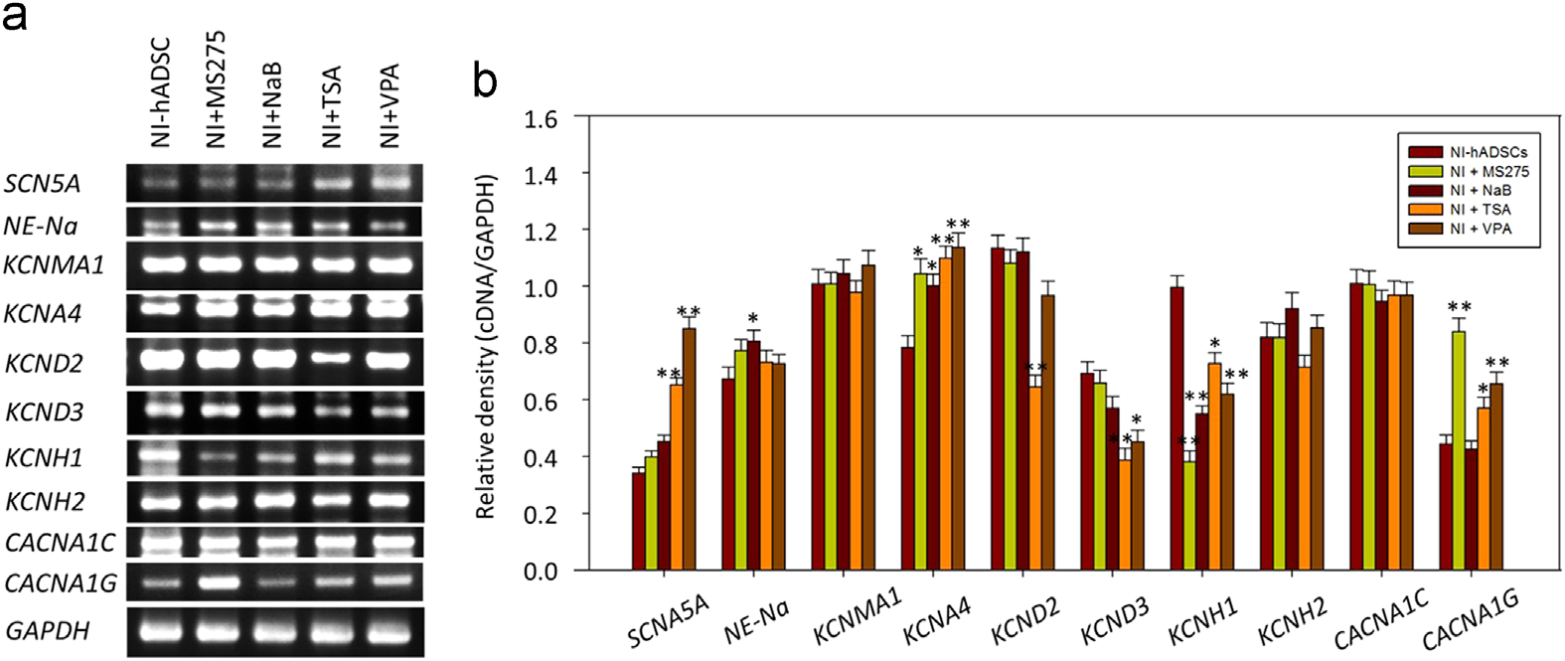
Modulation of ion channels-related gene expressions following treatment with the HDAC inhibitors. (a) RT-PCR was performed. (b) Histograms show quantification of gene expressions. GAPDH was used as a control. The RT-PCR assay was repeated five times independently in four different donor cells, and the representative data are shown. The expression level of each gene was normalized to that of GAPDH. *p<0.05, **p<0.01 compared with the expression from NI-hADSCs. NaB, sodium butyrate; TSA, trichostatin A; VPA, valproic acid; *SCN5A*, a TTX-insensitive Na^+^ channel; *NE-Na*, a TTX-sensitive Na^+^ channel; *KCNMA1*, a voltage- and calcium-dependent K^+^ channel; *KCNA4* & *KCND2* & *KCND3*, a human voltage-dependent K^+^ channel; *KCNH1* & *KCNH2*, a human ether-à-go-go K^+^ channel; *CACNA1C* & *CACNA1G*, a human voltage-dependent L-type Ca^2+^ channel (alpha 1C/1G subunit); *GAPDH*, glyceraldehyde 3-phosphate; RT-PCR, Reverse transcriptase-polymerase chain reaction.

In order to analyze neurogenic differentiation following HDCA inhibitors in hADSCs, we used four HDAC inhibitors; MS-275, NaB, TSA, or VPA. Morphological changes and NFL expressions were observed using phase contrast microscopy and immunofluorescence staining (Fig. 1). Furthermore, we measured the expressions of ion channel marker genes, which are responsible for outward and inward currents (Fig. 2). The primers used in this article are given in Table 1.

**Table 1:** Sequence of PCR primers.

**Gene**	**Forward (5′-3′)**	**Reverse (5′-3′)**
*SCN5A*	CCTAATCATCTTCCGCATCC	TGTTCATCTCTCTGTCCTCATC
*NE-Na*	GCTCCGAGTCTTCAAGTTGG	GGTTGTTTGCATCAGGGTCT
*KCNMA1*	ACAACATCTCCCCCAACC	TCATCACCTTCTTTCCAATTC
*KCNA4*	ACGAGGGCTTTGTGAGAGAA	CACGATGAAGAAGGGGTCAT
*KCND2*	ACCGTGACCCAGACATCTTC	CACTGTTTCCACCACATTCG
*KCND3*	GCCTCCGAACTAGGCTTTCT	CCCTGCGTTTATCAGCTCTC
*KCNH1*	TGGATTTTGCAAGCTGTCTG	GAGTCTTTGGTGCCTCTTGC
*KCNH2*	ACATCCTGCTTTTCGATTGG	CGGCTCTCTACCTGGCGTTG
*CACNA1C*	AACATCAACAACGCCAACAA	AGGGCAGGACTGTCTTCTGA
*CACNA1G*	CTGCCACTTAGAGCCAGTCC	TCTGAGTCAGGCATTTCACG
*GAPDH*	CATGACCACAGTCCATGCCATCACT	TGAGGTCCACCACCTGTTGCTGTA

## 2 Experimental design, materials and methods

### 2.1 Cell lines and reagents

HDAC inhibitor MS-275 (N-(2-aminophenyl)-4-[N-(3-pyridineyl-methoxycarbonyl) aminomethyl]-benzamide; Selleck Chemicals, Houston, TX, USA) was prepared as a stock solution in dimethylsulfoxide (DMSO; Sigma Chemical Co., St. Louis, MO, USA). In all the experiments, the final DMSO concentration was <0.1%. Other HDAC inhibitors NaB (Sigma Chemical Co.), TSA (Cell Signaling Technology Inc., Danvers, MA, USA), and VPA (Sigma Chemical Co.) were prepared as stock solutions in medium.

We cultured hADSCs and differentiated them into NI-hADSCs following our previously published methods [2], [3]. Cells were obtained according to the guidelines established by the Ethics Committee at the Chonnam National University Medical School (IRB:I-2009-03-016). The cells were grown as adherent cultures in Dulbecco's modified Eagle's medium (DMEM; Hyclone, Logan, UT, USA) supplemented with 10% fetal bovine serum (FBS; Hyclone), 1% penicillin-streptomycin (Gibco BRL, Grand Island, NY, USA), and 0.2% amphotericin B (Gibco) in a 37 °C humidified incubator with 5% CO_2_. To induce neural differentiation, the cells were maintained in DMEM containing 1% FBS and supplemented with 100 ng/mL bFGF (Invitrogen Co., Carlsbad, CA, USA), for seven days, and then incubated in 10 µM forskolin (Sigma Chemical Co.) for the next seven days. The HDAC inhibitors; MS-275 (500 nM), NaB (10 µM), TSA (40 µM), or VPA (10 µM); were added individually during the 14-day neural induction.

### 2.2 Phase contrast microscopy

To observe the morphology of cells, the cells were cultured following HDAC inhibitor treatment. Pictures were taken using an inverted microscope (Axio Vert. A1, Carl Zeiss, Germany).

### 2.3 Immunocytochemistry

The immunocytochemical procedure was modified from our previously published study [2]. Cells were grown on gelatin-coated Aclar plastic coverslips for 2 weeks, fixed for 10 min with chilled-methanol (Burdick & Jackson, Muskegon, MI, USA), and blocked for 20 min with 0.5% Triton X-100 (Sigma Chemical Co.) and 10% normal goat serum (Vector Laboratories Inc., Burlingame, CA, USA) in PBS. Anti-neurofilament (NFL; Santa Cruz Biotechnology, Santa Cruz, CA, USA; 1:100) were incubate overnight for 4 ℃ and then added the Alexa 488-conjugated goat anti-mouse antibody (Molecular Probes, Invitrogen Co., CA, USA; 1:200), which was used as a secondary antibody, at room temperature for 1 h. Nuclei were stained with 4′,6-diamidino-2-phenylindole (DAPI; Molecular Probes; 1 µg/mL) to allow cell counting. Cells were observed using a Zeiss microscope and pictures were taken. Experiments were performed in triplicate, and the number of positive cells was randomly counted. To perform the quantitative analysis, the number of positive cells was counted in each acquired image by ImageJ 1.61 (NIH), and the ratio of the number of positive cells to the number of nuclei was analyzed for the antigen.

### 2.4 Reverse transcriptase-polymerase chain reaction (RT-PCR) analyses

Total RNA was prepared in TriZol (Molecular Research Center, Inc., Cincinnati, OH, USA), according to the manufacturer's instructions, and 1 µg of cDNA was synthesized with the M-MLV Reverse Transcriptase (Gibco BRL) for 90 min at 42 °C. The cDNA was amplified by 35 cycles of 94 °C for 1 min, appropriate annealing temperatures for each primer for 1 min, and 72 °C for 1 min, using the Ex-Taq polymerase (Takara Bio Inc., Shiga, Japan) on PCR machine (Takara Bio Inc.). The forward and reverse PCR oligonucleotide primers that were selected to amplify the cDNA are listed in Table 1 (Bioneer Co., Daejeon, Korea). RT-PCR products were verified by electrophoresis on 2% agarose gels (Sigma Chemical Co.) The amount of cDNA was normalized based on the level of the ubiquitously expressed glyceraldehyde 3-phosphate (*GAPDH*) to analyze the relative expression of mRNAs [1], [2], [3], [4], [5].

### 2.5 Statistics

The levels of mRNA expression were quantified by measuring the optical density of each band by computer-assisted densitometry (NIH Image analysis program, version 1.61). A one-way analysis of variance (ANOVA) test (Bonferroni *post-hoc* comparison) was performed to analyze the differences between groups, with *p*<0.05 being considered to indicate statistically significant data. All values are expressed as the mean±standard error of the mean (SEM).
