# Supplementary material for: Data for the effect of histone deacetylase inhibitors on voltage- and ligand-gated ion channel gene expression in neurogenic induced-human adipose tissue-derived mesenchymal stem cells
Source: Data Brief. 2018 Feb 27;17:1314–9. doi: 10.1016/j.dib.2018.02.058 (PMC5988444; doi:10.1016/j.dib.2018.02.058)
Supplement: Supplementary file 1 — Supplementary material [file mmc1.docx]

Conflict of Interest

The authors have declared that they have no conflict of interests for this work.
